# Clinical analysis of different intestinal reconstruction methods after primary cytoreductive surgery combined with rectal resection for advanced ovarian cancer

**DOI:** 10.3389/fonc.2025.1500042

**Published:** 2025-01-27

**Authors:** Huimin Wang, Xiaocen Li, Ying Jiang, Jinxin Chen, Rong Cao, Jingru Zhang

**Affiliations:** Department of Gynecology, Liaoning Cancer Hospital and Institute, Shenyang, China

**Keywords:** advanced ovarian cancer, intestinal resection, intestinal reconstruction, protective enterostomy, anastomotic leak

## Abstract

**Objective:**

To compare different intestinal reconstruction methods after intestinal resection for advanced ovarian malignancy.

**Methods:**

Retrospective data of patients with advanced ovarian malignancy were collected and then assigned into three groups: primary intestinal anastomosis, protective enterostomy and colostomy. General clinical characteristics, intraoperative findings and postoperative outcomes were compared between the three groups.

**Results:**

A total of 530 cases were included for final analysis. The colostomy group had a lower serum albumin level, larger volume of ascites, higher likelihood of multiple intestinal resections and lower likelihood of rectal resection, lower peritoneal cancer index, more intraoperative blood loss, transfusions and infusions, lower likelihood of optimal cytoreductive surgery and shorter interval time to chemotherapy than the other two groups (*p* < 0.05). The primary intestinal anastomosis group exhibited a larger blood transfusion volume, higher incidence rates of anastomotic leak and electrolyte disturbance, and longer times to first flatus, first feeding and drain removal than the other two groups (*p* < 0.05).

**Conclusions:**

Colostomy can be adopted for advanced ovarian cancer patients with a large ascites volume, hypoproteinemia, large intraoperative blood and fluid loss volumes, multiple intestinal resections, anastomoses located below the peritoneal reflection, high PCI and suboptimal cytoreductive surgery. For patients with good intraoperative and postoperative outcomes, one anastomosis, an anastomosis located above the peritoneal reflection, low PCI or optimal cytoreductive surgery, intestinal anastomosis can be carried out to restore the normal physiological function of the intestine. For patients with a large volume of ascites (≥500 mL), multiple anastomoses or an anastomosis located below the peritoneal reflection, intestinal anastomosis combined with protective enterostomy has an advantage over intestinal anastomosis alone.

## 1 Introduction

Ovarian malignancies are one of the three major types of gynecological malignancies. Due to their insidious onset, over 70% of patients have advanced disease at the time of diagnosis (1). The primary malignancy is frequently accompanied by extensive metastases to the pelvis and abdominal cavity. The metastasis rate to the intestine is approximately 50%, the metastasis rate to the small intestine is 26%–33%, and the metastasis rate to the colorectum is 30%–39% (2, 3). Pelvic and rectal metastasis and extensive invasion to the rectouterine pouch are the most common sites of spread. For this reason, combined ovarian and intestinal resection has been increasingly applied in these cases (4). This approach aims to achieve optimal cytoreductive surgery, which can significantly prolong the survival time and improve the patient quality of life. Due to the high volume of concomitant resections, increasing attention has been paid to the method of intestinal tract reconstruction following intestinal resection.

The options of intestinal tract reconstruction after intestinal resection include intestinal anastomosis, enterostomy and colostomy. The methods for intestinal anastomosis include anastomosis alone (primary anastomosis, after partial resection of the rectum, free of the colon, and the remaining rectum is anastomosed with the colon without tension or blood supply obstacles at the anastomosis) and anastomosis plus protective enterostomy. Intestinal resection and anastomosis may lead to many postoperative complications, including anastomotic leak, anastomotic bleeding, anastomotic stenosis and intraabdominal infection. Leakage is the most severe and challenging complication. According to reports, the incidence rate of colorectal anastomotic leaks is 3%–24% (1, 5–8). The incidence rate of anastomotic leaks in colorectal or ileal-rectal anastomoses is much higher than that of colo-colic anastomoses. Patients with clinical anastomotic leak are associated with worse survival compared with patients without anastomotic leak (9, 10), and anastomotic leaks lead to death in 6%–26% of patients with colorectal cancer (8). Therefore, ways to prevent and/or mitigate the occurrence of anastomotic leaks are continually being explored by surgeons. The concept of protective stoma was first proposed in the low anterior resection of rectal cancer (11). Protective ostomy can prevent anastomotic fistula by transferring feces. Some studies have shown that the occurrence of anastomotic leaks is an event of small probability, and, even if they occur, anastomotic leaks can be clinically cured in most patients through conservative treatment methods such as fasting, intravenous nutrition and adequate drainage. A previous study has shown that protective enterostomy can prevent the occurrence of anastomotic leaks during intestinal surgery for advanced ovarian cancer (12). However, it has also been shown that protective enterostomy is not associated with anastomotic leaks during intestinal surgery for advanced ovarian cancer (13). As a result, the clinical significance of protective enterostomy remains controversial. Meanwhile, it is impossible to accurately predict the occurrence of anastomotic leaks in patients after intestinal anastomosis. The study on the risk factors related to anastomotic leakage shows that, the univariate analyses showed that male sex, the distance from the anal verge, and a duration of operation ≥140 min were associated with an increased incidence of anastomotic leakage in colorectal cancer (14), but preoperative serum albumin level <30 mg/dl, multiple bowel resections and primary cytoreduction have been identified as risk factors of anastomotic leakage in ovarian cancer (15). Because the biological behavior of ovarian cancer and colorectal cancer is inconsistent, the judgment of anastomotic leakage and which patients need protective ostomy cannot be completely based on intestinal cancer.

Enterostomies can be classified as either permanent or temporary. An example of a permanent ostomy is an end colostomy resulting from an abdominal-perineal resection in which the anus is excised through radical surgery for a low rectal malignancy. Temporary ostomies are generally protective enterostomies and usually ileostomies. Permanent enterostomies are generally adopted in circumstances where the anus cannot be preserved through radical surgery due to the low position of a malignant rectal tumor. However, in patients with ovarian malignancies complicated by intestinal metastasis, the lesions are located above the peritoneal reflection (in the rectum, rectouterine pouch, and sigmoid colon in most cases). Because the Douglas pouch serves as a line of defense, prohibiting tumor invasion beyond the peritoneum and infiltration into extraperitoneal tissues, colorectal anastomosis may be performed at the level of the mid-rectum or above the peritoneal reflection, and intestinal anastomosis or colostomy with preservation of the anus is possible. Therefore, a colostomy for ovarian cancer surgery is typically not a permanent enterostomy in the strictest sense, and like protective enterostomy, it can often be reversed. However, stoma reversal and postoperative complication rates may be higher in patients after protective enterostomy, while the quality of life of patients with protective enterostomy is poorer than that of patients with colostomy. Therefore, the selection, efficacy and clinical significance of different intestinal tract reconstruction methods after intestinal resection for advanced ovarian cancer are worthy of in-depth exploration.

In this work, the clinical data and follow-up data of patients with advanced ovarian cancer who underwent intestinal resection were retrospectively collected. The differences in clinical efficacy among different intestinal tract reconstruction methods were analyzed.

## 2 Methods

### 2.1 Study design

Data from patients with advanced ovarian malignancy undergoing enterectomy in the Department of Gynecology of Liaoning Cancer Hospital and Institute from January 2013 to December 2020 were retrospectively collected. The enrolled patients were grouped by intestinal tract reconstruction method as follows: primary intestinal anastomosis alone (n = 242), intestinal anastomosis + protective enterostomy (protective loop ileostomy) (n = 57) and colostomy (end-colostomy after Hartmann procedure) (n = 231) (**Table 1**). All intestinal resections and intestinal tract reconstructions were performed by experienced surgeons who each perform >50 intestinal resections per year. The intestinal anastomosis alone group was then subdivided into two groups based on whether anastomotic leaks occurred, and the groups were subanalyzed.

**Table 1:** Study flow chart.

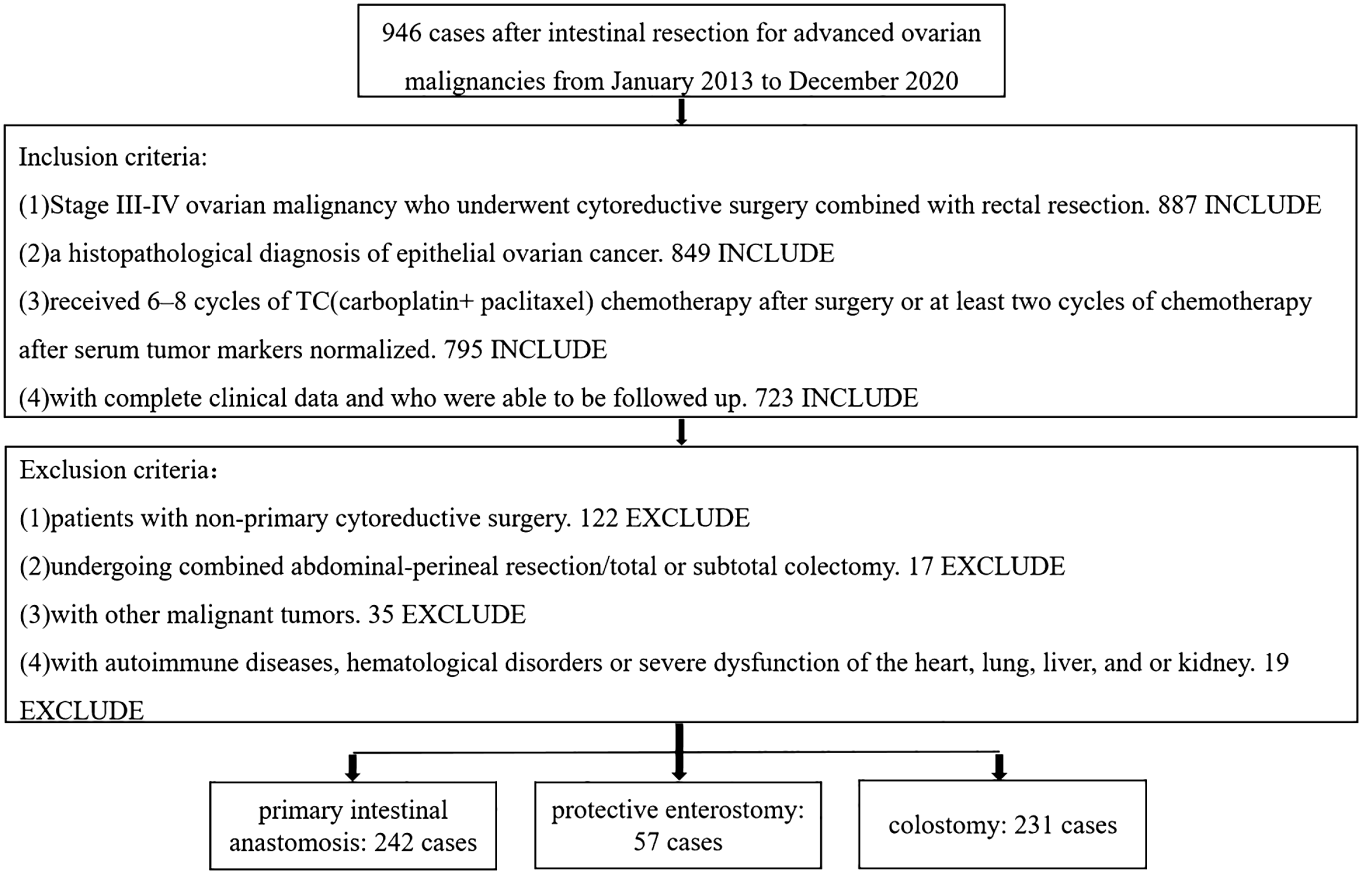

### 2.2 Data collection

All clinical datasets were complete, including age, body mass index (BMI), neoadjuvant chemotherapy, diabetes, stage, histologic subtype, differentiation, ascites, blood biochemical indexes, tumor markers, operation time, number and site of intestinal resection, peritoneal cancer index (PCI), level of cytoreduction achieved [complete (R0), optimal <1 cm (R1) and suboptimal (R2) (16)], complications, postoperative recovery, adjuvant therapy and stoma reversal. The final follow-up date was 31 December 2021. Medical information reviews, hospital follow-up and telephone follow-up were conducted at the same time point to ensure that the survival outcome at follow-up was accurate. After surgery, all patients were followed-up every 3 months for 2 years, and then every 6 months thereafter. Follow-up evaluations included survival status, recurrence time, recurrence site (local pelvic recurrence or distant recurrence), time of death (if applicable), and cause of death (tumor-related deaths or non-tumor deaths, if applicable). The study and its protocols were approved by the research ethics committee of Liaoning Cancer Hospital & Institute (No. 20220315G).

### 2.3 Statistical analysis

Data analysis was carried out with SPSS 26 software (IBM, Chicago, IL, USA). Continuous variables were analyzed by analysis of variance (ANOVA). The Chi squared test or Fisher’s Z correction exact test were used to compare categorical variables. OS and DFS were calculated by the Kaplan–Meier method and compared using the log-rank test. Multivariate analysis was performed using the logistic regression model. Results are presented as hazard ratios (HRs) with 95% confidence intervals (95% CIs). P values <0.05 were considered statistically significant.

## 3 Results

### 3.1 Preoperative status

The serum albumin level in the colostomy group was lower than in the other two groups (p < 0.05). No significant differences were noted in age, BMI, diabetes, stage, histologic subtype, differentiation, blood biochemical indexes (red blood cells, white blood cells, platelets, hemoglobin (HB), prealbumin, D-dimer (DD)), tumor markers (CA125, HE4, CA199) in the three groups (all p > 0.05) (**Table 2**).

**Table 2:** Comparison of preoperative status between the three cohorts.

Characteristic	Primary intestinal anastomosis (n=242)	Protective enterostomy (n=57)	Colostomy (n=231)	P
Age (year)	59.19 ± 8. 58	58.47 ± 9.17	60.13 ± 9.34	0.342
BMI (kg/m2)	22.84 ± 3.06	23.52 ± 3.02	23.29 ± 3.11	0.155
Neoadjuvant chemotherapy	69	15	76	0.543
Diabetes	22	3	31	0.120
Stage				0.875
III	198	47	183	
IV	44	10	38	
Differentiation				0.727
high-medium	25	5	19	
low	217	52	212	
Histologic subtype				0.637
serous	222	54	216	
other	20	3	15	
Blood biochemical indexes				
red blood cell (*10^9/L)	4.24 ± 0.50	4.36 ± 0.54	4.22 ± 0.43	0.116
white blood cell (*10^9/L)	6.61 ± 3.32	6.30 ± 1.60	6.43 ± 2.47	0.675
platelet (*10^9/L)	325.24 ± 116.36	318.18 ± 140.27	319.76 ± 133.44	0.869
HB(g/L)	125.12 ± 10.88	121.32 ± 8.31	124.61 ± 16.88	0.164
prealbumin(g/L)	197.71 ± 80.07	190.81 ± 57.69	183.55 ± 63.00	0.095
albumin(g/L)	41.14 ± 4.56	40.65 ± 3.80	39.18 ± 3.85	<0.001
DD (ug/L)	2.81 ± 4.92	2.49 ± 1.97	2.82 ± 1.90	0.805
Tumor markers				
CA125(U/ml)	1776.77 ± 2348.39	1586.56 ± 1919.42	2219.26 ± 3103.25	0.149
HE4(pmol/L)	946.08 ± 1462.94	820.98 ± 786.08	833.03 ± 868.19	0.702
CA199(U/ml)	58.24 ± 152.07	34.72 ± 77.32	52.97 ± 189.88	0.702

### 3.2 Intraoperative findings

The proportion of patients with optimal cytoreductive surgery was significantly higher in the intestinal anastomosis + protective enterostomy and intestinal anastomosis alone groups than in the colostomy group (87.72%, 85.13% *vs.* 74.46%). No statistically significant difference was found between the intestinal anastomosis + protective enterotomy group and the intestinal anastomosis alone group. The volume of ascites was significantly larger in the colostomy group than in the intestinal anastomosis + protective enterostomy and intestinal anastomosis alone groups (P = 0.032). No significant difference was found between the intestinal anastomosis + protective enterostomy and the intestinal anastomosis alone groups (p > 0.05). The value of PCI was significantly larger in the colostomy group than in the intestinal anastomosis + protective enterostomy and intestinal anastomosis alone groups (P < 0.001). No significant difference was found between the intestinal anastomosis + protective enterostomy and the intestinal anastomosis alone groups (p > 0.05). The rate of only rectal resection was the lowest but multiple resection was the highest in the colostomy group compared to the other two groups (P < 0.001). The proportions of patients with multiple intestinal segments resected and with rectal resections below the peritoneal reflection (17) were the largest in the colostomy group, followed by the intestinal anastomosis + protective enterostomy and intestinal anastomosis alone groups (56.08% *vs.* 26.32%, 14.46%, and 71.86% *vs.* 26.32%, 13.63%), with significant differences among the three groups. However, no significant difference was found between the intestinal anastomosis + protective enterostomy and the intestinal anastomosis alone groups. Intraoperative blood loss, blood transfusion and infusion volumes were significantly larger in the colostomy group than in the intestinal anastomosis + protective enterostomy and intestinal anastomosis alone groups. No significant difference was found between the intestinal anastomosis + protective enterostomy and intestinal anastomosis alone groups (p > 0.05). No significant difference was found in operation time between the three groups (p > 0.05) (**Table 3**).

**Table 3:** Comparison of intraoperative findings between the three cohorts.

Characteristic	Primary intestinal anastomosis (n=242)	Protective enterostomy (n=57)	Colostomy (n=231)	P
Ascites(ml)	884.83 ± 1153.29	775.44 ± 873.73	1100.13 ± 1005.11	0.032
Type of bowel resection				<0.001
Rectum	58	9	0	
Rectum-Colon	149	33	148	
Multiple	35	15	83	
Intestinal resection* number				0.017
1	207	42	148	
≥2	35	15	83	
Intestinal anastomosis				
ileocolonic	23	12	44	
small intestine-small intestine	9	3	15	
colon-colon	3	0	24	
sigmoid-rectal	157	45		
above peritoneal reflection	132	32		
below peritoneal reflection	25	13		
descending colon -rectum	27	3		
above peritoneal reflection	26	3		
below peritoneal reflection	1	0		
rectum-rectum	58	9		
above peritoneal reflection**	51	7		
below peritoneal reflection	7	2		
Rectum resection site				<0.001
above peritoneal reflection	209	42	65	
below peritoneal reflection	33	15	166	
Peritoneal Cancer Index				<0.001
	10.69 ± 6.35	13.57 ± 5.49	18.95 ± 4.81	
Cytoreduction				<0.001
R0	125	39	73	
R1	81	11	99	
R2	36	7	59	
Intraoperative blood loss(ml)	541.32 ± 350.47	489.47 ± 227.32	830.52 ± 669.98	<0.001
Intraoperative blood transfusion(ml)	752.07 ± 641.31	901.75 ± 709.18	1031.39 ± 705.57	<0.001
Intraoperative infusion volume(ml)	2214.67 ± 870.68	2023.68 ± 1193.86	2535.50 ± 1035.89	<0.001
Operation time(h)	4.98 ± 1.17	4.94 ± 1.77	4.97 ± 1.07	0.991

*Intestinal resection number of primary intestinal nastomosis and protective enterostomy equal to the number of anastomoses.

**Peritoneal reflection: The peritoneum covered in front of the middle and upper rectum folds forward, forming a rectocele, which divides the rectum into two parts, namely, the upper peritoneal rectal section and the lower extraperitoneal rectal section (17).

### 3.3 Postoperative outcomes

A total of 26 (10.43%) cases of anastomotic leaks were found in the intestinal anastomosis alone group, with statistically significant differences between the groups. The three groups showed no statistically significant differences in the rates of anastomotic bleeding and surgical site infection. The intestinal anastomosis alone group exhibited a significantly higher rate of intraabdominal infection than the intestinal anastomosis + protective enterostomy group (11.16% *vs.* 1.75%). The incidence of hypoproteinemia was the highest in the colostomy group, followed by the intestinal anastomosis alone and intestinal anastomosis + protective enterostomy groups (84.42% *vs.* 70.66%, 57.89%, respectively), and the differences were statistically significant. The incidence of anemia was significantly lower in the intestinal anastomosis + protective enterostomy group than in the intestinal anastomosis alone and colostomy groups (38.60% *vs.* 63.22%, 63.64%). No significant difference was noted between the intestinal anastomosis alone and colostomy groups. The rate of electrolyte disturbance was significantly higher in the intestinal anastomosis alone group than in the intestinal anastomosis + protective enterostomy and colostomy groups (21.90% vs. 8.77%, 6.93%). No significant difference was observed between the intestinal anastomosis + protective enterostomy and colostomy groups.

The time to first flatus and first feeding were the shortest in the intestinal anastomosis + protective enterostomy group, followed by the intestinal anastomosis alone and colostomy groups. The time to postoperative drain removal was shorter in the intestinal anastomosis + protective enterostomy and colostomy groups than in the intestinal anastomosis alone group. The incidence rate of grade III–IV myelosuppression was significantly higher in the intestinal anastomosis + protective enterostomy group than that in the intestinal anastomosis alone and colostomy groups (35.09% *vs.* 19.42%, 9.09%). The time to induction of chemotherapy was significantly shorter in the colostomy group than that in the intestinal anastomosis alone and intestinal anastomosis + protective enterostomy groups, and the differences were statistically significant (**Table 4**).

**Table 4:** Comparison of postoperative outcomes between the three cohorts.

Characteristic	Primary intestinal anastomosis (n=242)	Protective enterostomy (n=57)	Colostomy (n=231)	P
Anemia(HB<80g/L)	153	22	147	<0.001
Hypoproteinemia(albumin<25g/l)	171	33	195	<0.001
Electrolyte disturbance	53	5	16	<0.001
Surgical site infection	2	0	0	0.539
Intraabdominal infection	27	1	17	0.056
Anastomotic bleeding	3	2	2	0.664
Anastomotic leak	26	0	0	<0.001
First flatus time (d)	5.14 ± 1.60	3.60 ± 1.19	4.74 ± 2.16	<0.001
First feeding time (d)	10.78 ± 6.57	5.49 ± 2.92	7.90 ± 2.94	<0.001
Postoperative drain removal time (d)	13.38 ± 4.32	9.26 ± 3.49	9.66 ± 3.04	<0.001
Induction of chemotherapy time (d)	28.26 ± 14.93	28.26 ± 11.27	22.20 ± 10.39	<0.001
III/IV Side effects of chemotherapy	47	20	21	<0.001

### 3.4 Analysis of risk factors related to anastomotic leak

#### 3.4.1 Comparison of preoperative status and operation-related conditions between the two groups

The subdivision of the intestinal anastomosis alone group demonstrated that 10.74% (n = 26) of the cohort experienced anastomotic leak and 89.26% (n = 216) did not. Patients in the anastomotic leak group were older and more likely to have diabetes mellitus (23.08% *vs.* 7.41%). No significant differences were noted in BMI, neoadjuvant chemotherapy, diabetes, stage, histologic subtype, differentiation, blood biochemical indexes (red blood cells, white blood cells, platelets, HB, prealbumin, albumin, DD), CA125, HE4 and CA199 between the two groups (all p > 0.05) (**Table 5**).

**Table 5:** Comparison of general conditions and surgical conditions between the two groups.

Characteristic	Anastomotic leak (n=26)	Non-anastomotic leak (n=216)	P
Age (year)	63.00 ± 7.96	58.74 ± 8.55	0.016
BMI (kg/m2)	22.97 ± 3.26	22.81 ± 3.05	0.809
Neoadjuvant chemotherapy	9	61	0.500
Diabetes	6	16	0.009
Stage			0.079
III	18	180	
IV	8	36	
Differentiation			0.275
high-medium	1	23	
low	25	193	
Histologic subtype			0.890
serous	23	193	
other	3	23	
Blood biochemical indexes			
red blood cell(*10^9/L)	4.35 ± 0.67	4.23 ± 0.48	0.253
white blood cell(*10^9/L)	6.53 ± 1.76	6.62 ± 3.47	0.891
platelet(*10^9/L)	290.23 ± 86.66	329.45 ± 118.90	0.105
HB(g/L)	128.42 ± 12.89	124.73 ± 10.58	0.102
prealbumin(g/L)	290.23 ± 86.66	329.45 ± 118.90	0.105
albumin(g/L)	41.51 ± 5.62	41.10 ± 4.42	0.666
DD(ug/L)	2.71 ± 2.78	2.83 ± 5.12	0.907
Tumor markers			
CA125(U/ml)	2190.24 ± 3757.17	1698.44 ± 2089.53	0.356
HE4(pmol/L)	960.18 ± 1455.03	882.84 ± 1408.06	0.838
CA199(U/ml)	94.54 ± 169.75	42.76 ± 117.88	0.110
Ascites(ml)	1684.62 ± 1434.63	788.56 ± 1079.29	<0.001
Intestinal resection number			<0.001
1	17	191	
≥2	9	25	
Intestinal anastomosis			
ileocolonic	6	17	
small intestine-small intestine	4	5	
colon-colon	0	3	
sigmoid-rectal	18	139	
above peritoneal reflection	10	122	
under peritoneal reflection	8	17	
descending colon -rectum	3	24	
above peritoneal reflection	3	23	
under peritoneal reflection	0	1	
rectum-rectum	5	53	
above peritoneal reflection	3	48	
under peritoneal reflection	2	5	
Rectum resection site			<0.001
above peritoneal reflection	10	23	
under peritoneal reflection	16	216	
Peritoneal Cancer Index	12.28 ± 6.80	10.32 ± 6.22	0.232
Cytoreduction			0.066
R0	9	116	
R1	14	67	
R2	3	33	
Intraoperative blood loss(ml)	426.92 ± 232.48	552.58 ± 361.77	0.086
Intraoperative blood transfusion(ml)	753.85 ± 497.37	743.66 ± 656.89	0.939
Operation time(h)	4.10 ± 0.94	4.93 ± 1.05	0.104
Postoperative Complications			
Anemia(HB<80g/L)	17	136	0.809
Hypoproteinemia(albumin<25g/l)	18	163	0.489
Surgical site infection	2	0	<0.001
Intraabdominal infection	7	20	0.018
Anastomotic bleeding	1	2	0.205
Recto-vaginal fistulas	18	0	0.034
Secondary enterostomy	16	0	0.046
First flatus time (d)	5.12 ± 2.14	5.15 ± 1.53	0.917
First feeding time (d)	21.04 ± 15.09	9.56 ± 2.77	<0.001
Postoperative drain removal time (d)	22.71 ± 6.75	12.57 ± 3.16	<0.001
Induction of chemotherapy time (d)	47.04 ± 20.59	26.00 ± 12.36	<0.001
Course of normalize CA125			0.016
≤3	11	149	
4-8	7	39	
Not normal	8	28	
III/IV side effects of chemotherapy	6	41	0.618
Recurrence	18	104	0.042
Death	15	61	0.008

The volume of ascites, number of patients with multiple resections and/or with anastomoses below the peritoneal reflection were significantly higher in the anastomotic leak group than in the non-anastomotic leak group (33.33% *vs.* 10.68%, 38.46% *vs.* 9.50%). No statistically significant differences were found in PCI value, operation satisfaction, intraoperative blood loss, blood transfusion, infusion volumes and operation time between the two groups (p > 0.05) (**Table 5**).

The incidence rates of postoperative surgical site and intra-abdominal infection were significantly higher in the anastomotic leak group than in the non-anastomotic leak group (29.17% *vs.* 9.22%). The time to first feeding and postoperative drain removal was longer in the anastomotic leak group than in the non-anastomotic leak group. No statistically significant difference was found in anemia, hypoproteinemia, anastomotic bleeding and first flatus time between the two groups (p > 0.05) (**Table 5**).

#### 3.4.2 Prognosis

**Figure 1:**
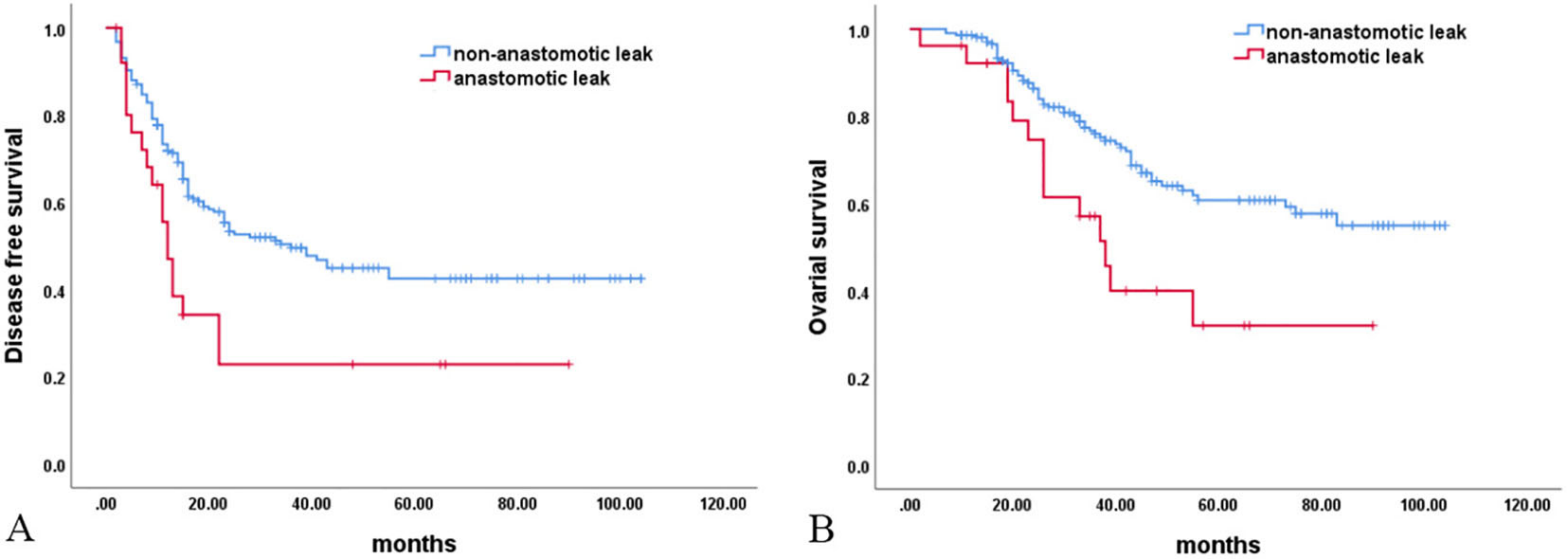
Survival analysis. **(A)** Disease-free survival (P = 0.005); **(B)** Overall survival (P = 0.006).

In the anastomotic leak group (n = 26), 18 cases (69.23%) of recto-vaginal fistulas were found. Sixteen (88.89%, 16/18) of these patients required a second enterostomy for their recto-vaginal fistulas. Fourteen (77.78%, 14/18) underwent a second procedure for their recto-vaginal fistulas. The interval time to the commencement of chemotherapy after surgery in the anastomotic leak group was significantly shorter than in the non-anastomotic leak group. The number of treatments to normalize tumor marker (CA125) levels was significantly lower in the non-anastomotic leak group than in the anastomotic leak group. In addition, 19 cases (73.08%) of recurrence and 15 (57.69%) deaths occurred in the anastomotic leak group. These rates were significantly higher than those in the non-anastomotic leak group [104 cases (48.15%) vs 61 cases (28.24%)]. Univariate Kaplan–Meier analysis demonstrated significantly shorter DFS and OS in the anastomotic leak group than in the non-anastomotic leak group (median time 12 *vs.* 36 months, 33 vs. 38 months). The incidence rate of grade III–IV side effects of chemotherapy was comparable between the two groups (**Table 6**, **Figure 1**).

**Table 6:** Comparison of prognosis between the two groups.

Characteristic	Anastomotic leak (n=26)	Non-anastomotic leak (n=216)	P
Recto-vaginal fistulas	18	0	0.034
Secondary enterostomy	16	0	0.046
Induction of chemotherapy time(d)	47.04 ± 20.59	26.00 ± 12.36	<0.001
Course of normalize CA125			0.016
≤3	11	149	
4-8	7	39	
Not normal	8	28	
III/IV side effects of chemotherapy	6	41	0.618
Recurrence	18	104	0.042
Death	15	61	0.008
Not stoma reversal	3	8	0.189

Multivariate logistic analysis was conducted on the factors associated with anastomotic leaks found in the univariate analysis (age, diabetes mellitus, ascites volume, number of intestinal segments resected, and intestinal resection site). Ascites, resection of >1 intestinal segments and intestinal anastomosis below the peritoneal reflection were independent risk factors for anastomotic leaks (**Table 7**).

**Table 7:** Multivariate logistic analysis of anastomotic leaks.

Characteristic	HR(95%CI)	P
Age (year)	0.531(0.133-2.126)	0.371
Diabetes	2.777(0.813-9.489)	0.103
Ascites	6.688(1.876-23.845)	0.003
>1 intestinal resection	3.700(1.332-10.279)	0.012
Intestinal anastomosis below the peritoneal reflection	4.030(1.480-10.971)	0.006

## 4 Discussion

In this study, the clinical data of advanced ovarian cancer patients undergoing colostomy, intestinal anastomosis alone and intestinal anastomosis + protective enterostomy were retrospectively analyzed. It was found that the colostomy group had more comorbidities, which was reflected by a high incidence of preoperative hypoproteinemia due to a poor nutritional status and a large ascitic volume. Additionally, high PCI and extensive lesions gave rise to a large range of intestinal resections and multi-segment intestinal resections and a lower number of rectal resections. The time of the first chemotherapy after permanent enterostomy was significantly shorter than that of intestinal anastomosis in this study. Because the prognosis of ovarian cancer is related to the satisfaction of surgery and chemotherapy, if satisfactory cytoreductive surgery cannot be achieved, immediate chemotherapy is likely to improve the survival time of patients (18, 19). While the recovery time of patients after intestinal anastomosis will be long and the commencement of chemotherapy after surgery will be delayed, which may affect the survival time of patients, suboptimal cytoreduction may be an influencing factor for surgeons that choose permanent enterostomy. Non-optimal cytoreductive surgery may be an important factor when the surgeon is considering permanent enterostomy. The incidence rate of anastomotic leaks after intestinal anastomosis alone was higher than after protective enterectomy and permanent enterectomy. In the case of anastomotic leaks, feces flows into the abdominal cavity through the leak, resulting in intraabdominal infection. During conservative treatment or secondary surgery for anastomotic leaks, patients experience prolonged fasting times, and thus, have increased rates of anemia and electrolyte imbalances as well as larger postoperative blood transfusion volumes. In cases of both protective enterostomy and permanent colostomy, feces do not travel through the intestinal anastomosis. As a result, the incidence rates of postoperative fasting and electrolyte disturbance are significantly reduced. In addition, the time to flatus is short, after which patients can begin to eat. Moreover, the postoperative recovery is quicker and the interval time to first chemotherapy is shorter. Consequently, the prognosis of patients is improved.

The data of 299 patients receiving intestinal resection and anastomosis were subjected to stratification analysis. Compared with the intestinal anastomosis group, the protective enterostomy group had higher optimal cytoreduction rates and numbers of intraoperative intestinal anastomoses and anastomoses located below the peritoneal reflection. They also had notably decreased rates of anastomotic leaks, anastomotic bleeding, intra-abdominal infections, anemia, hypoproteinemia and ion disturbances and shorter times to flatus and eating after surgery. A reason why the surgeon may choose protective enterostomy may be that patients with optimal cytoreduction have the best prognosis and the possibility of reversing the stoma is high.

Advanced ovarian malignancies exhibit different biological behaviors from malignant colorectal tumors. First, adverse patient factors, such as a poor nutritional status, a large volume of ascites, and extensive intraperitoneal metastasis are often found in patients with advanced ovarian malignancies. As a result, such patients have an increased rate of anastomotic leaks. Secondly, resection of the greater omentum and abdominopelvic cavity peritoneum are typically performed during surgery for advanced ovarian cancer. This weakens the wrapping and protective effects of the omentum and peritoneum on the anastomosis, thereby increasing the risk of anastomotic leaks. Lastly, the standard procedure of cytoreductive surgery for ovarian cancer typically includes hysterectomy, and thus recto-vaginal fistulas, severe abdominal infection and other fatal complications may develop once anastomotic leaks occur, delaying chemotherapy and affecting the prognosis of patients. Therefore, the choice of the intestinal tract reconstruction method after intestinal resection for ovarian malignancy cannot be generalized to that of colorectal cancer. In the present study, 26 cases (10.74%) of anastomotic leaks were detected in the intestinal anastomosis alone group. This is significantly higher than the incidence found in anastomoses for intestinal cancer (i.e., 2%) (20). Eighteen cases of recto-vaginal fistulas were found in the anastomotic leak group, accounting for 69.23% (18/26) of the cohort, of which 16 cases required secondary enterostomy, corresponding to 88.89% (16/18) of the recto-vaginal fistula patient group. Fourteen cases (78%) required a second operation for recto-vaginal fistulas. As a result, patients had a prolonged hospitalization, increased cost, poor prognosis and shortened survival time due to the significantly prolonged interval time to chemotherapy after surgery. This further proves the importance of protective enterostomy for intestinal resection and anastomosis in advanced ovarian cancer.

Protective enterostomy enables early feeding and early chemotherapy (21, 22); however, it also has many disadvantages. In contrast to large intestinal stomas, small intestinal stomas may affect the absorption of nutrients. Additionally, the incidence rates of other enterostomy complications, such as dehydration, electrolyte disturbance and dermatitis in stomas, is high. A second operation is required 3–6 months later to reverse the stoma, which can cause psychological and physical trauma to patients and increases medical costs (23). For patients with colorectal cancer, stoma repair is conducted within 3 months after surgery in most cases (24, 25). The response rate of first-line chemotherapy for ovarian cancer exceeds 70%, thus emphasizing the importance of initiating chemotherapy promptly following surgery for ovarian malignant tumors. It is generally recommended to commence chemotherapy within 2 weeks after surgical recovery of gastrointestinal function, preferably not exceeding a duration of 4 weeks. Timing of cytotoxic treatment [≤ 28 days *vs.* >28 days] was a significant prognostic factor for overall survival in multivariate analysis (19).Stoma reversal is delayed in most patients with ovarian cancer to facilitate chemotherapy. In this study, 11 patients (19.30%) in the protective enterostomy group ultimately ended up with permanent stomas, as they could not undergo stoma reversal due to primary platinum chemotherapy resistance, cancer progression during chemotherapy and/or short-term recurrence. Although studies have shown that 90% of patients with malignant intestinal tumors do not benefit from protective enterostomy (5), if enterostomy is rashly implemented in all advanced ovarian cancer patients undergoing intestinal resection, this may place an unnecessary burden on some patients and lead to unnecessary surgery and trauma. Therefore, it is particularly important to master the indications for protective enterostomy during intestinal anastomosis for advanced ovarian cancer.

Furthermore, in this study, a subgroup analysis was carried out on patients with intestinal anastomosis alone to further investigate the indications for preventive enterostomy. Univariate analysis demonstrated that age, diabetes mellitus, a large ascitic volume, multiple anastomoses and anastomoses located below the peritoneal reflection were implicated in the development of anastomotic leaks. The results of further multivariate logistic analysis revealed that an ascitic volume ≥500 mL, multiple anastomoses and anastomoses located below the peritoneal reflection were independent risk factors for anastomotic leaks. A previous study has shown that ascitic volume is an independent risk factor for poor prognosis in ovarian malignancy (26). In this study, we demonstrated for the first time that an ascitic volume ≥500 mL was an independent risk factor for anastomotic leaks after intestinal anastomosis for ovarian malignancy. This may be due to the fact that the inflammatory microenvironment and extracellular matrix remodeling caused by TNF-αand MMP-2 in ascitic fluid of patients with ovarian cancer may be related to poor healing of intestinal anastomosis (27). Most patients with colorectal cancer have one anastomosis, so the relationship between the number of intestinal segments resected and the occurrence of anastomotic leaks in colorectal cancer remains poorly studied (28). The results of this study showed that resection of multiple intestinal segments was an independent risk factor for anastomotic leaks after intestinal resection and anastomosis for ovarian malignancy. This is mainly attributed to the following factors. First, multiple anastomoses suggest longer operation times, which is also an independent risk factor for anastomotic leaks. According to previous studies on the association between anastomotic location and anastomotic leaks in colorectal cancer, anastomoses close to the anus are a high-risk factor for leaks (28, 29). A ‘safe’ anastomosis should have low tension. In patients with ovarian malignancies, the low location of rectal anastomoses and the resection of multiple intestinal segments can result in increased anastomotic tension, giving rise to an increased risk of anastomotic leaks. Moreover, compared to colorectal cancer patients, those with advanced ovarian cancer also have an increased risk of infection due to hysterectomy. A previous study has reported that pelvic and intra-abdominal infection is also a high-risk factor for anastomotic leaks (30). In this study, the incidence rate of postoperative intra-abdominal infection in the anastomotic leak group was significantly higher than that in the non-anastomotic leak group.

In this study, there were few cases in the anastomotic leak group, so it was necessary to further increase the sample size to make the results more reliable. Additionally, some imaging data were incomplete due to the long review time and the fact that imaging data of some patients came from other hospitals. The complexity of the disease was not analyzed by the peritoneal metastasis score and Suidan CT score, which is also the focus of follow-up research. Some patients with advanced ovarian cancer with primary resistance to platinum chemotherapy or rapid disease progression, even if intestinal anastomosis and protective enterostomy are performed, may not be suitable for stoma reversal. Additionally, protective small intestinal enterostomy is inferior to permanent large intestinal enterostomy in nutrient absorption. Direct permanent enterostomy may have an advantage over intestinal anastomosis and protective enterostomy for such patients. Hence, the identification of patients with primary resistance to platinum and refractory disease and the selection of intestinal resection and reconstruction methods warrant further investigation.

## Data Availability

The original contributions presented in the study are included in the article/supplementary material. Further inquiries can be directed to the corresponding author.
